# Propranolol monotherapy versus propranolol plus prednisone for diffuse infantile hepatic haemangioma: a randomized clinical trial

**DOI:** 10.1007/s12519-026-01070-1

**Published:** 2026-07-14

**Authors:** Yu-Ru Lan, Zi-Xin Zhang, Lin-Yu Yang, Min Yang, Tong Qiu, Kai-Zhi Zhang, Jiang-Yuan Zhou, Xue Gong, Xue-Peng Zhang, Chun-Shui He, Qiang Peng, Fang Hou, Wei Shan, Ping-Qian Bao, Chun-Chao Xia, Si-Yuan Chen, Yi Ji

**Affiliations:** 1 Division of Oncology, Department of Pediatric Surgery, and Rare Diseases Center, West China Hospital of Sichuan University, 37 Guo-Xue-Xiang, Chengdu 610041, China; 2 Pediatric Intensive Care Unit, Department of Critical Care Medicine, West China Hospital of Sichuan University, 37 Guo-Xue-Xiang, Chengdu 610041, China; 3 Department of Vascular Surgery, Hospital of Chengdu University of Traditional Chinese Medicine, Chengdu 610032, China; 4 Department of Pediatric Surgery, Chengdu Women and Children’s Central Hospital, Chengdu 610073, China; 5 Department of Pediatric Surgery, Sichuan Academy of Medical Sciences and Sichuan Provincial People’s Hospital, Chengdu 610072, China; 6 Department of Pediatric Surgery, Sichuan Women and Children’s Hospital, Chengdu 610036, China; 7 Department of Pediatric Surgery, Leshan Women and Children’s Hospital, Leshan 614099, China; 8 Department of Radiology, West China Hospital of Sichuan University, 37 Guo-Xue-Xiang, Chengdu 610041, China

**Keywords:** Efficacy, Infantile hepatic haemangioma, Prednisone, Propranolol, Safety

## Abstract

**Background:**

Oral propranolol, with or without corticosteroids, has been shown to benefit diffuse infantile hepatic haemangioma (IHH); randomized trial evidence is lacking. We aimed to evaluate the efficacy and safety of propranolol monotherapy versus propranolol plus prednisone for the treatment of problematic IHHs to determine whether oral prednisone provided an added benefit to propranolol.

**Methods:**

We conducted an open-label, multicenter, randomized controlled trial across six referral centers in China from July 2019 to November 2023 (clinicaltrials.gov registration: NCT03331744). Infants with problematic diffuse IHH were assigned (1:1) to oral propranolol alone or propranolol plus a short course of prednisone. The primary endpoint was the proportion of patients who achieved a lesion response at week 4 on serial ultrasound. Analyses followed the intention-to-treat principle.

**Results:**

Forty-five patients were included in this study (propranolol, *n* = 22; propranolol plus prednisone, *n* = 23). Four weeks following treatment, lesion response rates were higher with propranolol plus prednisone than with propranolol alone [21/23 (91.3%) vs. 13/22 (59.1%); difference: 32.2%; 95% confidence interval (CI) = 6.9%–53.5%; *P* = 0.007]. Compared with monotherapy, combination therapy yielded higher lesion response rates during weeks 1–3, more rapid stabilization of thyroid-stimulating hormone levels during weeks 2–4, and a greater overall volumetric response at month 6 (91.3% vs. 63.6%; difference: 27.7%; 95% CI = 3.1%–49.3%; *P* = 0.026). Total adverse events were similar between the groups, and no grade 3–4 adverse events were observed.

**Conclusions:**

Addition of prednisone to propranolol in patients with diffuse IHH markedly improved lesion response rates. Propranolol plus prednisone represents a valid treatment for diffuse IHH.

**Graphical abstract:**

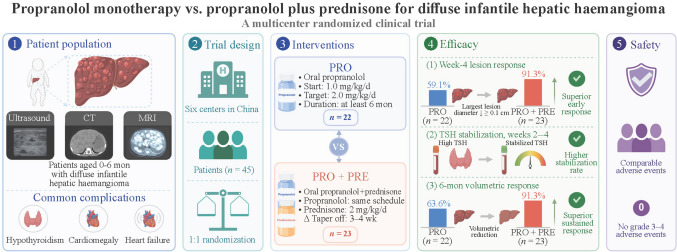

## Introduction

Infantile hepatic haemangiomas (IHHs) are benign hepatic vascular tumours that occur in the paediatric population. IHHs share the same growth patterns as cutaneous infantile haemangiomas (IHs) and express glucose transporter-1. IHHs are categorized into two subtypes: multifocal and diffuse. Diffuse IHHs differ from multifocal IHHs in that diffuse IHHs result in total or extensive replacement of the liver with innumerable vascular lesions [1, 2]. A small minority of multifocal IHHs can continue to proliferate and ultimately evolve into diffuse IHHs if they are unrecognized and/or untreated [3]. Evidence suggests that diffuse IHHs display serious clinical presentations and can result in severe complications such as hypothyroidism, high-output congestive cardiac failure, hepatic failure, abdominal compartment syndrome and respiratory distress [4–6]. Clinically, patients with diffuse IHHs have a higher risk of mortality than those with multifocal IHHs.

Prompt therapeutic intervention for diffuse IHHs is both needed and necessary. The American Academy of Pediatrics (AAP) guidelines for the treatment of IH suggest that IHHs are best treated with pharmacologic therapy [7]. Treatments with corticosteroids, cyclophosphamide, vincristine and/or interferon, whether used alone or in combination, have been recommended for the treatment of diffuse IHHs. However, these standard treatment regimens are inadequate because responses to these pharmacological therapies are variable and unpredictable [8, 9]. In addition, the long-term use of these pharmacological therapies may be associated with significant and sometimes irreversible side effects.

Recent studies have demonstrated that treatment of IHHs with oral propranolol (with or without corticosteroids) results in marked resolution of tumour lesions and attenuation of associated life-threatening complications [10–14]. However, these findings are based on case reports and retrospective studies. In addition, the combination of propranolol with corticosteroids has been shown to result in faster responses and improved safety in cutaneous IH treatment [15]. Therefore, we conducted this multicenter, open-label, randomized controlled trial (RCT) to assess the efficacy and safety of propranolol versus propranolol plus prednisone in patients with diffuse IHHs.

## Methods

### Ethical approval

This trial protocol was approved by the ethics committee at all study institutions (approval number: 2017414). The trial was performed in accordance with the principles of the Declaration of Helsinki. Written informed consent was obtained from the parents/guardians of all patients included. This trial is registered with clinicaltrials.gov under the identifier of NCT03331744.

### Trial design

This study is an open-label, multicenter RCT comparing propranolol and propranolol plus prednisone for the treatment of patients with diffuse IHHs. The trial was designed to verify whether propranolol plus prednisone was superior to propranolol monotherapy for the treatment of diffuse IHHs. Six tertiary medical centers collaborated in this trial from July 2019 to November 2023. Data were obtained from each institution and sent to West China Hospital of Sichuan University for analysis.

### Randomization and masking

Randomization was conducted centrally at West China Hospital of Sichuan University using a computer-generated random sequence. The participants were randomly assigned at a 1:1 ratio to receive propranolol monotherapy or propranolol plus a short-term application of prednisone. Guardians and clinicians who managed the study participants were aware of the treatment assignments. Researchers and the statistical team who assessed study endpoints were blinded to the participant allocation group.

### Study participants

Patients with diffuse IHHs were identified based on clinical features and typical ultrasound (US) findings along with computed tomography (CT) and/or magnetic resonance imaging (MRI). Inclusion criteria were: (1) patients aged between 0 and 6 months; (2) a diagnosis of diffuse IHH requiring systemic therapy; and (3) parental consent. Exclusion criteria were: (1) contraindications to the use of β-blockers; (2) a history of treatment with propranolol or other β-blockers; (3) exposure to corticosteroids, vincristine, sirolimus, embolization or any other treatments for IHH within one week before enrolment; and (4) an inability to follow the study protocol. Diffuse IHHs were defined as the near-total replacement of the normal liver parenchyma with innumerable haemangioma lesions [1, 16].

### Study intervention

The propranolol treatment schedule was at least 6 months. Treatment consisted of propranolol monotherapy or a combination of propranolol and prednisone. All patients received oral propranolol at a dosage of 1.0 mg/kg/day divided into 2 doses daily for 3 days, which was then increased to 2 mg/kg/day divided into 2 doses daily from day 4. Prednisone was administered orally at 2 mg/kg once daily in the morning. Oral prednisone was tapered and stopped within 3–4 weeks if patients had satisfactory responses (e.g., normalization of thyroid and cardiac function). The criteria for early discontinuation of the trial included the following: treatment was deemed ineffective (clinical and radiologically progressive disease), parent/guardians refusal to continue, or the investigator considered it necessary to terminate treatment because of concomitant illness or any grade ≥ 4 adverse events (AEs). At the end of the 6-month treatment period, patients were allowed to continue if they experienced a benefit from propranolol and the investigator determined no safety concerns. All patients were scheduled for regular follow-up visits for 12 months after enrolment.

At enrolment the following tests were performed for all patients, a physical examination, full blood count, coagulatory function, liver and renal function, thyroid function, an electrocardiogram and echocardiogram. IHHs were examined using US with colour Doppler by trained sonographers. US was performed weekly for one month, then every other week for two months, and then monthly for three months. Enhanced CT scans or MR images were acquired sequentially at baseline and at 6 months. Physical examination and routine laboratory tests were performed during study protocol visits and also between visits if needed. All US, CT and MR images were evaluated by an independent expert panel. On the basis of the clinical presentations and laboratory tests, each patient was assigned to one or more categories of IHH-related symptoms and complications.

Supportive care was recommended for patients with severe complications: thyroid hormone replacement to treat hypothyroidism, and digoxin and diuretics to treat high-output cardiac failure. Need for any additional IHH-specific therapy or intervention (e.g., embolization) was considered treatment failure.

### Study outcomes

Primary and secondary outcomes were established before commencing the trial. The primary endpoint was the difference between the two groups in terms of the proportion of patients who achieved a lesion response at week 4. A lesion response was defined as a decrease in the maximal diameter (≥ 0.1 cm) of the largest lesion after treatment. The investigators compared changes in lesions after treatment with the baseline via a US examination. Maximum lesion diameter is a commonly used endpoint for measuring the severity and treatment response of vascular tumours [17, 18].

The secondary outcomes included between-group differences in lesion response during weeks 1–3, the proportion of patients who experienced thyroid-stimulating hormone (TSH) stabilization during weeks 1–4, the overall volumetric response at month 6 and treatment compliance. To assess changes, the investigators compared lesion volume measurements 6 months after treatment with the baseline lesion volume measurements via CT/MRI. The volumetric responses were categorized as follows: complete lesion involution (100%), nearly complete lesion involution (decrease in lesion volume < 100% and ≥ 75%), good partial lesion involution (decrease in lesion volume < 75% and ≥ 50%), fair partial lesion involution (decrease in lesion volume < 50% and ≥ 20%), lesion stability (< 20% decrease or < 20% increase in lesion volume), or further lesion enlargement (increase in lesion volume ≥ 20%). An overall volumetric response included partial, nearly complete and complete involution.

Heart rate, blood pressure, and blood glucose levels were measured at baseline and then at 1, 2, 4 and 8 hours after the initiation of propranolol treatment. The presence of any AEs, such as bradycardia, hypotension and hypoglycaemia was recorded. Safety data were also collected via physical examinations and routine laboratory studies during scheduled visits. Severity of AEs were graded according to the Common Terminology Criteria for Adverse Events, version 4.0 (CTCAE v4.0). Patient withdrawal from the trial because of ineffectiveness and/or severe AEs was considered treatment failure.

### Statistical analysis

Forty patients (20 in each group) were required to identify a statistically significant difference between the two groups of ≥ 15% (90% for propranolol plus prednisone versus 75% for propranolol monotherapy) in the primary outcome with a 2-tailed *α* error of 0.05, with a power of 80%. Considering withdrawal of consent and loss to follow-up, we planned to recruit at least 44 patients. The primary endpoint was analysed on an intention-to-treat basis. We used Fisher’s exact test or a Chi-square test to analyse categorical variables. We used a Mann‒Whitney *U* test to analyse continuous variables. We used generalized linear mixed models to compare the difference in changes in TSH levels between baseline and 4 weeks between the two groups. SPSS 23.0 for Windows (SPSS Inc., Chicago, IL, USA) was used to perform all statistical analyses.

## Results

### Patient demographics and haemangioma characteristics

**Fig. 1:**
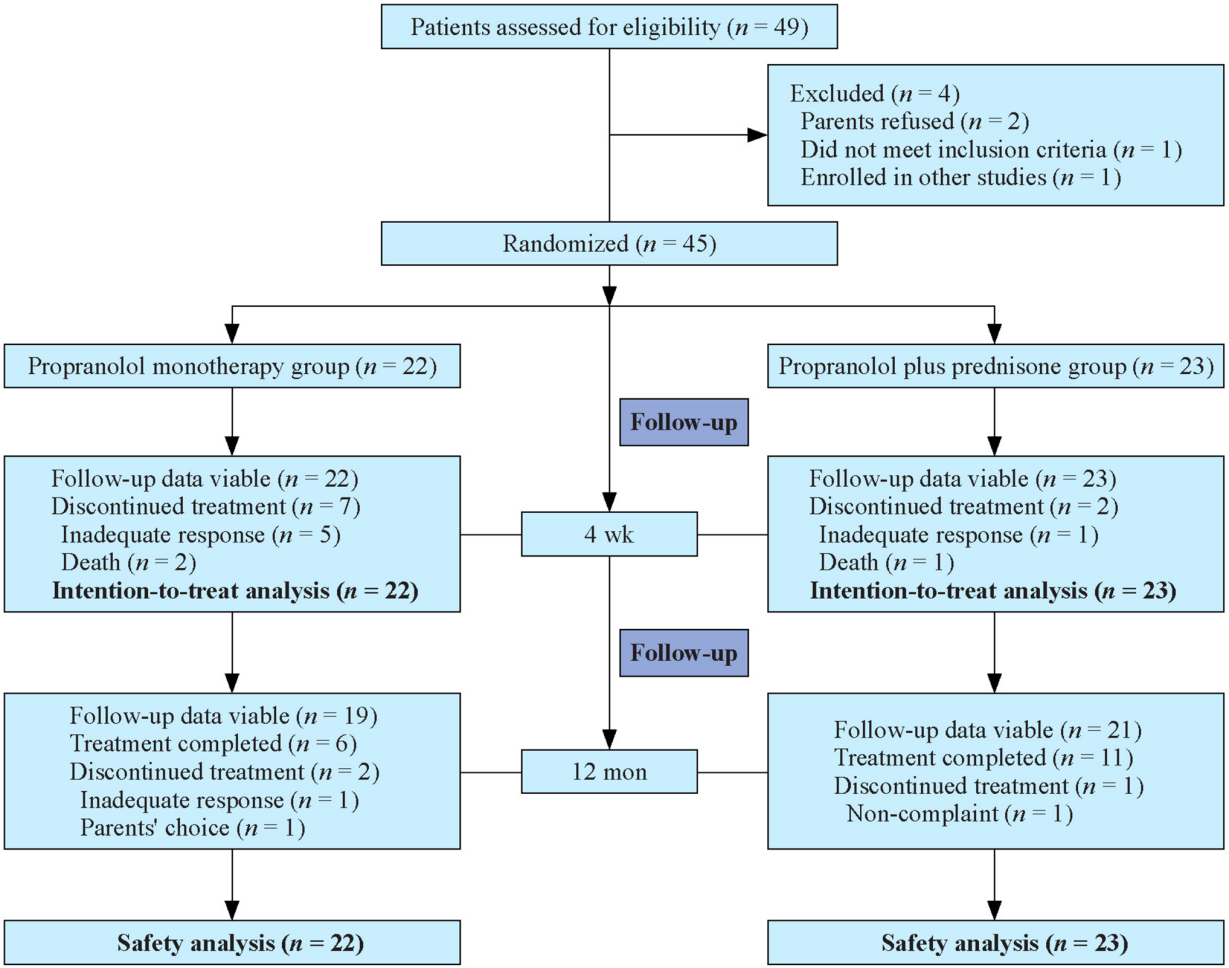
Consort flow diagram

A total of 49 patients were included (Fig. 1). Amongst these patients, 45 were eligible and randomly assigned to receive propranolol monotherapy (*n* = 22) or propranolol plus prednisone (*n* = 23). Mean age at baseline was 36.1 days (range: 1.0–175.0 days). Indications for treatment included symptoms (e.g., failure to thrive and abdominal distention) and/or complications (e.g., hypothyroidism and high-output congestive cardiac failure). The most common complications were hypothyroidism (97.8%) and cardiomegaly (77.8%). The two groups were similar with respect to demographics and baseline disease characteristics (Table 1).

**Table 1:** Comparison of demographic and clinical characteristics between the propranolol monotherapy and the combination treatment groups

Parameters	Propranolol (*n* = 22)	Propranolol plus prednisone (*n* = 23)	*P*
Sex, *n* (%)			0.672^a^
Female	15 (68.2)	17 (73.9)	
Male	7 (31.8)	6 (26.1)	
Age at baseline (d)			0.952^b^
Mean (range)	36.5 (1.0–175.0)	35.7 (1.0–169.0)	
Median (IQR)	14.5 (5.3–52.8)	16.0 (6.0–60.0)	
Gestational age, *n* (%)			1.000^c^
Born prematurely	5 (22.7)	5 (21.7)	
With cutaneous IH, *n* (%)			0.673^a^
Yes	14 (63.6)	16 (69.6)	
Maximal tumor dimension (cm)			0.632^b^
Mean (range)	2.4 (1.0–3.5)	2.5 (1.0–3.8)	
Median (IQR)	2.4 (2.0–3.0)	2.5 (2.0–3.0)	
Hypothyroidism, *n* (%)			0.489^d^
Yes	21 (95.5)	23 (100.0)	
TSH (μU/mL)			0.847^b^
Mean (range)	53.0 (11.6–151.0)	50.6 (14.3–152.6)	
Median (IQR)	42.6 (22.2–78.9)	33.2 (23.4–75.2)	
Cardiomegaly, *n* (%)			0.862^a^
Yes	13 (59.1)	13 (56.5)	
Heart failure, *n* (%)			0.641^a^
Yes	9 (40.9)	11 (47.8)	

*IH* infantile haemangioma, *TSH* thyroid-stimulating hormone, *IQR* interquartile range. ^a^*P* value using Chi-square test; ^b^*P* value using Mann‒Whitney *U* test; ^c^*P* value using Fisher’s exact test; ^d^*P* value using Pearson Chi-square test

### Efficacy

All 45 patients were included in the efficacy analyses. Ten patients withdrew from the study prematurely because of lack of efficacy [36.4% in the propranolol monotherapy group compared with 8.7% in the combination treatment group; difference: 27.7%; 95% confidence interval (CI) = 3.1%–49.3%]. Three patients died from disease progression [two (9.1%) patients in the propranolol monotherapy group and one (4.3%) patient in the combination treatment group]. Thirty-five patients completed the 6-month treatment period (Fig. 1). In the combination group, the mean duration of prednisone therapy was 9.3 weeks (range: 5.5–11.0 weeks) in patients who completed six months of treatment.

For the primary outcome, the proportion of patients who achieved a lesion response at week 4 was greater in the propranolol plus prednisone group than in the propranolol monotherapy group (91.3% vs. 59.1%; difference: 32.2%; 95% CI = 6.9%–53.5%; *P* = 0.007) (Table 2).

**Table 2:** Analysis of the primary and secondary outcomes

Outcomes	Propranolol (*n* = 22)	Propranolol plus prednisone (*n* = 23)	*P*
Primary outcome			
Proportion of patients achieving a lesion response at week 4	13 (59.1)	21 (91.3)	0.007^a^
Secondary outcome			
Proportion of patients achieving a lesion response during weeks 1–3			
Week 1	3 (13.6)	11 (47.8)	0.023^b^
Week 2	6 (27.3)	16 (69.6)	0.005^a^
Week 3	10 (45.5)	19 (82.6)	0.009^a^
Proportion of patients achieving TSH stabilization during weeks 1–4^c^			
Week 1	4 (19.0)	10 (43.5)	0.111^b^
Week 2	5 (23.8)	14 (60.9)	0.017^b^
Week 3	8 (38.1)	17 (73.9)	0.017^a^
Week 4	12 (57.1)	20 (87.0)	0.027^a^
Overall volumetric response (6 mon)	14 (63.6)	21 (91.3)	0.026^a^
Complete lesion involution	0 (0.0)	0 (0.0)	N/A
Nearly complete lesion involution	3 (13.6)	7 (30.4)	0.284^b^
Good partial lesion involution	5 (22.7)	8 (34.8)	0.514^b^
Fair partial lesion involution	6 (27.3)	6 (26.1)	0.928^a^

Values are presented as *n* (%). *TSH* thyroid-stimulating hormone, *N/A* not available. ^a^*P* using Chi-square test; ^b^*P* value using Fisher’s exact test; ^c^in both groups, only patients with hypothyroidism were included in the analysis

Compared with propranolol monotherapy, the combination treatment resulted in a higher lesion response rate during weeks 1 to 3. In addition, from 2 to 4 weeks after treatment, significantly greater proportions of patients achieved TSH stabilization in the combination treatment group than in the monotherapy group. Moreover, after six months of treatment, a greater proportion of patients in the combination group had an overall volumetric response than those in the propranolol monotherapy group (91.3% vs. 63.6%; difference: 27.7%; 95% CI = 3.1%–49.3%; *P* = 0.026).

### Safety and tolerance

There were no serious treatment-emergent adverse events (CTCAE v4.0 grade 3 or 4). Common AEs included agitation and sleep disturbance (Table 3). Hypotension was noted in one patient in the propranolol monotherapy group; this was transient and without sequelae. The patient continued propranolol treatment without changing regimen. The total numbers of AEs and individual AEs were comparable between the two groups.

**Table 3:** Comparison of treatment related adverse events in the propranolol monotherapy and the combination treatment groups^a^

Known adverse events associated with trial drugs^b^	Propranolol (*n* = 22)	Propranolol plus prednisone (*n* = 23)	*P*
Agitation	3	4	1.000^d^
Sleep disturbance	2	4	0.665^d^
Diarrhea	2	2	1.000^d^
Cool extremities	2	1	0.608^d^
Hypotension	1	0	0.489^d^
Growth disability	0	1	1.000^d^
Serious adverse event^c^	0	0	N/A
Total	10	12	0.652^e^

*N/A* not available. ^a^The safety population included all the patients who received at least one dose of the treatment under investigation; values are presented as numbers; ^b^adverse events were assessed using the Common Terminology Criteria for Adverse Events, version 4.0 (CTCAE v4.0); ^c^serious adverse events were defined as any of the following identified during the 24 weeks of treatment: grade ≥ 3 adverse events, persistent hypotension, persistent bradycardia, or intolerable central nervous system-related adverse effects; ^d^*P* value using Fisher’s exact test; ^e^*P* value using Chi-square test

## Discussion

Diffuse IHHs are associated with a higher risk of complications. In the present study, all patients were symptomatic and/or presented with complications. Clinical manifestations were comparable to those reported in previous studies. Hypothyroidism and cardiomegaly were extremely common. Given the serious potential morbidity, the importance of screening for potential complications or comorbidities in patients with diffuse IHHs cannot be overemphasized.

Because of its efficacy, durability and favourable tolerability, propranolol is considered the first-line alternative treatment to corticosteroid-based regimens for cutaneous IHs. In the present study, however, when used as a single agent, a lesion response was seen in only 59.1% of patients. Compared with propranolol monotherapy, a combination of propranolol and prednisone yielded a better treatment response with a similar safety profile. The 4.3% mortality rate in the combination group is among the most favourable outcomes reported in the literature (mortality ranged from 16.7% to 37.5%) [5, 19, 20]. Indeed, we verified that propranolol in combination with short-term prednisone was a useful and well-tolerated therapy for patients with diffuse IHHs. Patients with severe IHH should be treated aggressively with propranolol plus corticosteroids, and propranolol monotherapy is usually not recommended. If deterioration ensues during propranolol treatment, an alternative should be considered.

Because changes in lesion size and symptoms/complications may vary across patients, we used several measurements to accurately assess the overall improvement in IHH activity after treatment. US has been shown to be a practical, reliable and convenient non-invasive tool and is particularly suitable for measuring changes in IHH lesions after treatment [2]. Lesion response rates in the combination treatment group were significantly higher than those in the propranolol monotherapy group. We provide further clinical evidence that early aggressive treatment should be considered when diffuse IHHs are symptomatic and/or complicated. Additional data supporting our findings for the primary outcome were that TSH levels normalized more rapidly in the combination treatment group.

In parallel with objective lesion response assessment by US, the volumetric response, measured using enhanced CT or MRI, is a widely accepted outcome measurement for evaluating changes in the size of vascular tumours [21]. We observed a significantly higher overall volumetric response in the combination treatment group as the trial continued (six months after treatment), suggesting that the therapeutic effects of propranolol on diffuse IHHs are largely persistent.

Previous studies have suggested that a higher dose of propranolol (3 mg/kg/day) can yield a better response than a lower dose (1 mg/kg/day) in cutaneous IHs [22]. In this trial, we used 2 mg/kg/day oral propranolol, which was recommended by the European expert consensus group and the clinical practice guidelines from the AAP [7, 23]. However, although the lesion response at week 4 in the combination group was 91.3%, more than two-fifths of patients in the propranolol monotherapy group responded poorly to this regimen (2 mg/kg/day). Whether the administration of propranolol at higher doses than we used would be more effective is unknown. In addition, due to clinical heterogeneity and rarity of IHHs, we were unable to analyse potential risk factors for a poor response.

The mechanism by which propranolol plus prednisone induces better responses is a topic of great interest. Our findings suggest that propranolol plus prednisone may provide greater therapeutic benefit than propranolol monotherapy in patients with diffuse IHH. Interestingly, previous studies have revealed that propranolol and corticosteroids exert anti-haemangioma effects via distinct mechanisms. Propranolol is thought to promote pericyte-mediated vasoconstriction, inhibit angiogenesis and disrupt haemodynamic force-induced endothelial cell survival [24, 25]. Corticosteroids also have multiple mechanisms of action, including inhibition of vasculogenesis in haemangiomas [26].

Prolonged corticosteroid use may produce growth retardation, hypertension, metabolic abnormalities, and immunosuppression [27]. In the present trial, all documented AEs were mild. Indeed, we did not observe severe AEs related to the application of propranolol with prednisone. In addition, combining propranolol with short-term prednisone treatment did not increase overall adverse effects. The safety profile of propranolol plus prednisone in patients with diffuse IHHs was comparable to published data from patients receiving short-term treatment with corticosteroids [28].

Our study has several limitations. First, our study was an open-label trial. Both local site investigators and families knew the group in which our patients were placed. These factors might limit the internal validity and results of a trial. However, the researchers and statistical team who assessed study outcomes were blinded to group assignments and were unaware of patient clinical information. In addition, lesion and volume response assessments were objective and involved standardized image measurements and independent readings. Second, our findings are limited by small sample size. However, diffuse IHHs are rare, and our study is the largest prospective trial to date. Moreover, it is currently the only RCT on this disease. Third, in both groups, patients received supportive treatments that may have confounded the results. This recurring issue has been noted in trials of patients with critical diseases such as diffuse IHHs. However, despite this confounder, our data were positive. Finally, as noted, we were unable to perform a subgroup analysis, and our study had insufficient power for the prediction of an inadequate response.

In conclusion, this trial demonstrated that propranolol plus prednisone resulted in significant clinical benefits compared with propranolol monotherapy in patients with diffuse IHHs. The incidence of typical corticosteroid-related side effects was not increased by additional short-term prednisone. These data support future larger studies of propranolol plus corticosteroids as a potential therapy for patients with diffuse IHHs.

## Data Availability

The datasets used and/or analyzed during the current study are available from the corresponding author upon reasonable request.
